# Overexpression of *StTCP10* Alters Tuber Number and Size in Potato (*Solanum tuberosum* L.)

**DOI:** 10.3390/plants14091403

**Published:** 2025-05-07

**Authors:** Tingting Wang, Xinyue Chen, Shuangshuang Li, Ping Wang, Yongbin Wang, Binquan Huang

**Affiliations:** 1 State Key Laboratory for Conservation and Utilization of Bio-Resources in Yunnan, School of Agriculture, Yunnan University, Kunming 650500, China; ynwtt@mail.ynu.edu.cn (T.W.); 18724974815@163.com (X.C.); lishuangshuang0707@163.com (S.L.); wangping_b52s@itc.ynu.edu.cn (P.W.); 2 Southwest United Graduate School, Kunming 650500, China

**Keywords:** potato, *StTCP10*, tuber size, tuber number, yield

## Abstract

Potato (*Solanum tuberosum* L.), cultivated worldwide for its nutrient-rich underground tubers, represents a crucial staple crop whose yield is primarily determined by both tuber number and tuber size. TCP transcription factors, especially TCP containing miR319 binding sites, play pivotal roles in plant growth and development, yet their functions in potato tuber number and size remain largely unexplored. In this study, we systematically identified 32 *TCP* genes in potato harboring the conserved TCP domain, among which six were predicted to contain binding sites for Stu-miR319. Semi-quantitative experiments revealed that *StTCP10* exhibited the highest expression levels in stolons, swollen stolons, and tuber tissues compared to other *StTCP* genes containing miR319 binding sites. To elucidate its biological function, we generated *StTCP10*-overexpressing transgenic potato lines through *Agrobacterium*-mediated genetic transformation. Phenotypic analysis demonstrated that overexpression of *StTCP10* reduced tuber number per plant while enhancing tuber size, with no significant change in total yield. These findings reveal that *StTCP10* with Stu-miR319 binding sites plays a critical role in tuber size and mediates the trade-off between tuber size and number, providing novel insights into the molecular breeding aimed at improving tuber size.

## 1. Introduction

Potato (*Solanum tuberosum* L.) is a vital staple crop, with tubers serving as nutrient-rich underground storage organs. Tuber development typically involves four stages: stolon initiation, stolon elongation, subapical stolon swelling, and tuber enlargement [1]. Tuber number and size are key agronomic traits governing final yield in potato, highlighting the biological significance of these traits in yield formation. Under optimal growth conditions, tuber number typically exhibits a positive correlation with total yield [2]. Additionally, tuber size and uniformity significantly influence yield. Larger and uniformly sized tubers are highly preferred in the market, enhancing the commercial value of potatoes, which contributes to reduced postharvest losses and indirectly improves overall yield [3]. Therefore, understanding the genetic mechanisms underlying tuber number and size is essential for the potato industry.

TCP (teosinte branched1/cycloidea/proliferating cell factor1 and 2) proteins are a class of plant-specific transcription factors exclusively found in angiosperms [4]. The name “TCP” derives from the four founding members of this family: TEOSINTE BRANCHED 1 (TB1) in *Zea mays* [5,6], CYCLOIDEA (CYC) in *Antirrhinum majus* [7,8], and PROLIFERATING CELL FACTORS 1 and 2 (PCF1 and PCF2) in *Oryza sativa* [9]. All TCP family members share a highly conserved basic helix–loop–helix (bHLH) motif of 59 amino acids, which is responsible for DNA binding and protein–protein interactions [9,10]. Based on the differences in amino acid sequences, TCP transcription factors are classified into two subfamilies, class I TCPs and class II TCPs. Class I TCPs are also known as TCP-P class, such as PCF1 and PCF2 in rice [11], while Class II TCPs are further divided into CYC/TB1 TCP and CINCINNATA (CIN) TCP based on sequence variations within the TCP domain [12].

Accumulated evidence highlights the critical roles of TCP family members in plant growth and development [13,14]. In *Arabidopsis thaliana*, TCP proteins regulate diverse developmental processes. Viola et al. found that *AtTCP11* mutant plants exhibit abnormal pollen development [15], while *AtTCP14* and *AtTCP1* regulate inflorescence shoot apex growth and plant height and promote embryonic development [16]. *AtTCP16* is expressed during microspore formation and participates in male gamete development [17], whereas the *AtTCP23* gene modulates flowering rhythm and development [18]. Additionally, *TCP18* (*BRC1*) and *TCP12* (*BRC2*) suppress lateral branch outgrowth, with *AtBRC1* mutants displaying increased branching compared to wild-type plants [19,20,21]. Similar roles have been observed in other species: the loss of TB1 function in maize leads to increased branching [22], RNAi-mediated knockdown of *SlBRC1b* in tomato results in enhanced shoot branching [23], and the *CsBRC1* mutant leads to increased axillary buds [24]. Furthermore, *SlTCP26* negatively regulates auxin signaling to promote lateral branch development [25]. In potato, Nicolas et al. reported that the *BRANCHED1a* (*BRC1a*) gene controls the growth of aerial and underground lateral shoots [26], while the loss of function of *BRANCHED1b* (*BRC1b*) leads to ectopic aerial tuber formation and reduced underground tuberization [27]. *StTCP1* is involved in activating the meristem, controlling branching, and inducing secondary tuber growth and enlargement [28,29]. Additionally, Wang et al. reported that *StTCP15* affects the ratio between abscisic acid and gibberellic acid to modulate potato tuber dormancy and germination [30], and *StTCP23*-silenced plants showed stunted, curled leaves, and increased disease susceptibility compared to the control [31,32]. Furthermore, TCP transcription factor StAST1 interacts with both StSP6A and StABL1, leading to the suppression of activated tuberization complex (aTAC) formation and consequent modulation of maturity timing in potato [33].

Numerous studies have demonstrated that some CIN-class *TCP* genes contain miR319 binding sites, enabling post-transcriptional regulation by miR319 [34,35]. In *Arabidopsis*, the expression of CIN-class TCP transcription factors was regulated by miR319, with the *jaw-D* mutant showing suppressed expression of *TCP2*, *TCP3*, *TCP4*, *TCP10*, and *TCP24*, leading to undifferentiated leaf cells and severe leaf flatness [34,36]. Ori et al. found that LANCEOLATE (*LA*), a CIN-class *TCP* gene regulated by miR319, influences compound leaf formation of tomato [37]. Similarly, Nag et al. revealed that the overexpression of miR319 leads to narrow petals and abnormal anther development in *Arabidopsis thaliana* plants [38]. In cotton, *GhTCP4* is negatively regulated by miR319, resulting in thinner cell walls in cotton fibers [39]. Moreover, the miR319/TCP4 module affects tomato resistance to root knot nematodes by regulating the expression of jasmonic acid (JA) synthesis genes and the accumulation of endogenous JA [40]. In *Acer palmatum*, *ApTCP2* regulates leaf morphology, flowering periods, and leaf senescence through miR319-mediated mechanisms [41]. *TCP* genes harboring miR319 binding sites play pivotal roles in leaf development, flower organ formation, pollen development, aging, circadian rhythm, and hormone signal transduction [14]. However, their biological function in potato has not yet been elucidated.

To elucidate the regulatory role of *StTCP* genes harboring miR319 binding sites in tuber size and number, in this study, *StTCP10* genes containing miR319 binding sites were characterized, and *StTCP10*-overexpressing transgenic plants were generated and planted for phenotypic evaluation. The findings demonstrate that *StTCP10* genes containing Stu-miR319 binding sites enhance tuber size and mediate the trade-off between tuber size and number, providing novel insights into molecular breeding aimed at improving tuber size.

## 2. Results

### 2.1. Genome-Wide Identification of StTCP Genes in Potato

**Figure 1:**
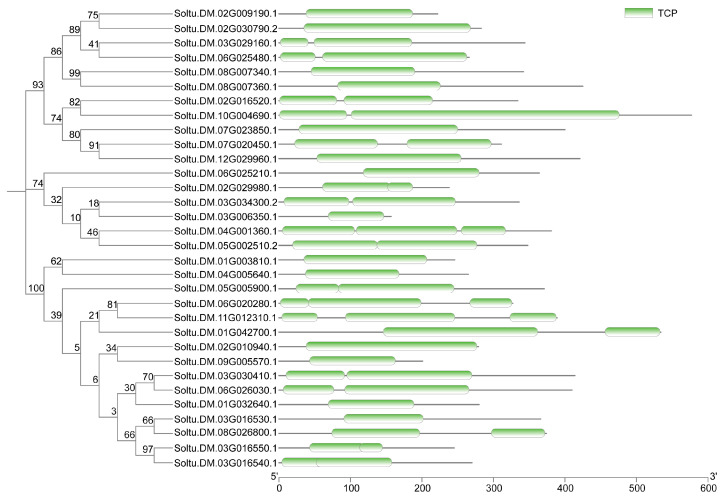
Analysis of intraspecific clustering and conserved domain of *StTCP* genes in potato.

**Figure 2:**
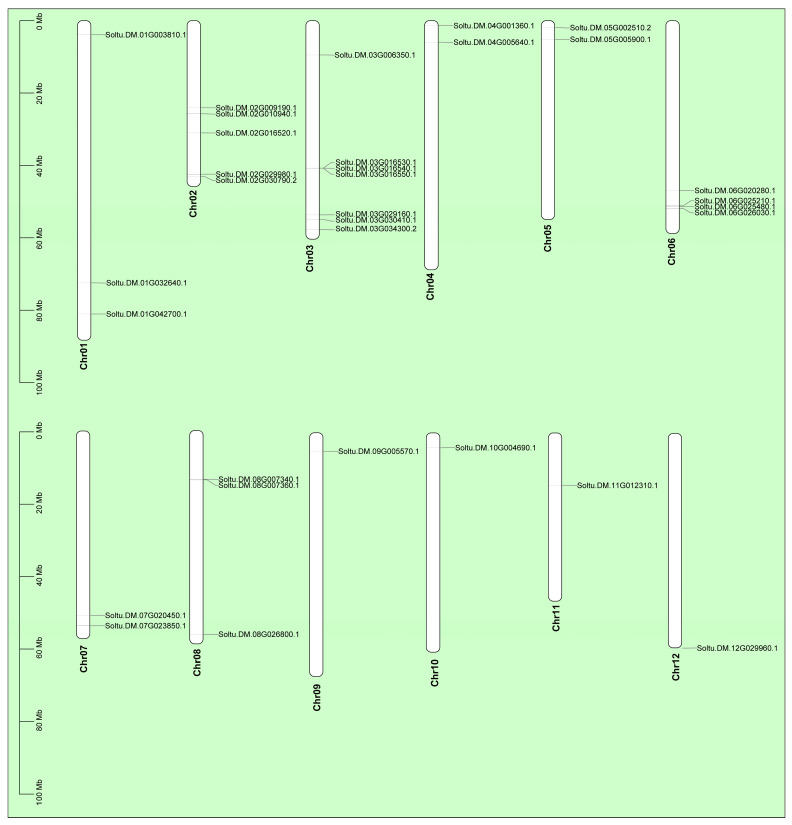
Chromosomal localization of *StTCP* genes.

Through the alignment of whole-genome protein sequences between potato and *Arabidopsis* TCP families, we systematically identified 32 potato TCP members harboring typical TCP domains, following exclusion of sequences lacking this characteristic domain (Figure 1). Chromosomal localization analysis revealed an uneven arrangement of these genes across 12 chromosomes (Figure 2). Notably, Chromosome 3 harbored the highest number of *TCP* genes (seven genes), while chromosomes 9 to 12 contained only one *TCP* gene each. Further analysis demonstrated that potato TCP proteins exhibit remarkable size diversity, with lengths varying from 157 amino acids (the most compact) to 577 amino acids (the most extended member) (Table S1). The variation in amino acid residue numbers suggest potential structural and functional diversity among these proteins, providing a foundation for further in-depth studies on the specific biological roles of each member.

### 2.2. Tissue-Specific Expression of StTCP Genes

**Figure 3:**
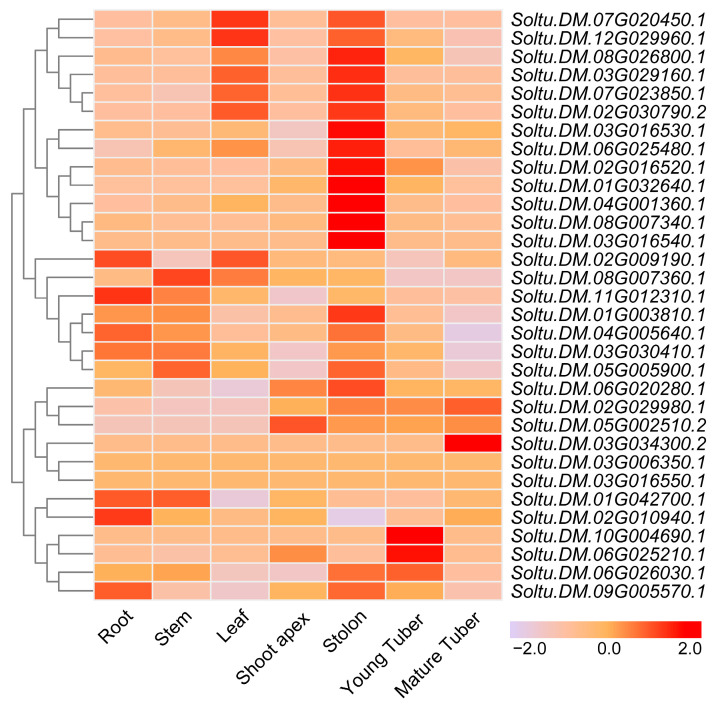
Expression pattern analysis of *StTCPs* in different tissues of potato.

The gene expression profiles in different tissues facilitate the understanding of their biological functions. To elucidate the function of *TCP* genes in potato development, we analyzed their spatial expression patterns across seven distinct tissues (root, stem, leaf, shoot apex, stolon, young tuber, and mature tuber) using RNA-Seq expression data from the Spud database. The results showed that members of the potato *TCP* family exhibited distinct expression patterns across these tissues (Figure 3). Notably, twenty-two genes displayed higher expression during the stolon stage, while seven genes were predominantly expressed during the young tuber stage (Figure 3). These distinct expression patterns strongly suggest potential roles for different TCP members in regulating tuber initiation and development processes.

### 2.3. Analysis of the Binding Sites of miR319/TCPs

**Figure 4:**
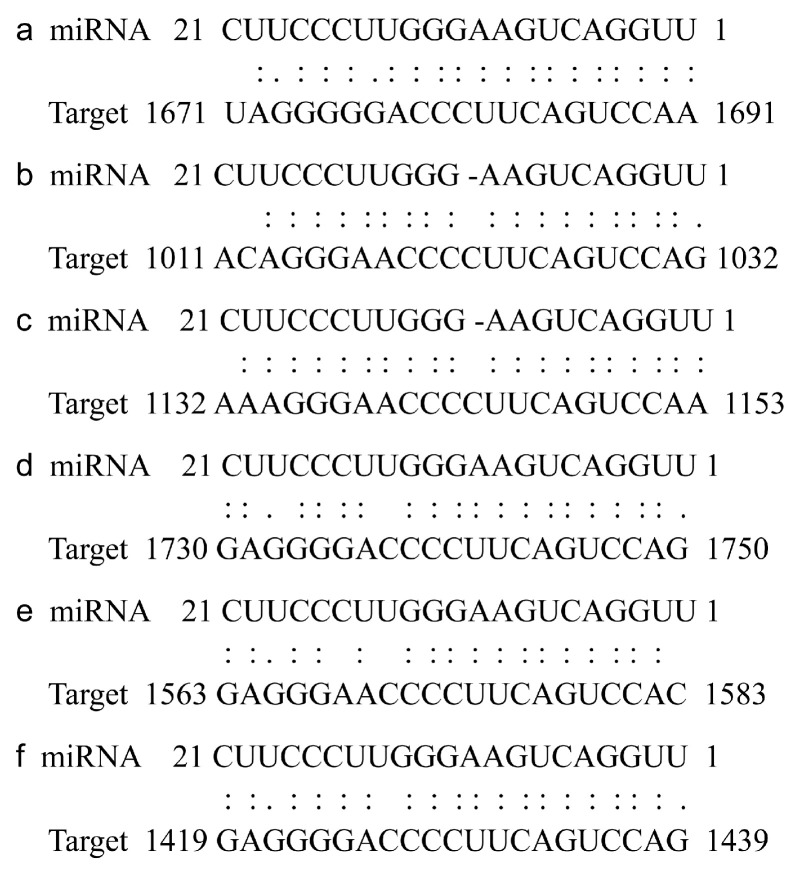
Analysis of *StTCP* genes with Stu-miR319 binding sites in potato. (**a**) *Soltu.DM.08G007340*. (**b**) *Soltu.DM.02G016520*. (**c**) *Soltu.DM.07G020450*. (**d**) *Soltu.DM.07G023850* (*StTCP10*). (**e**) *Soltu.DM.10G004690*. (**f**) *Soltu.DM.12G029960*.

**Figure 5:**
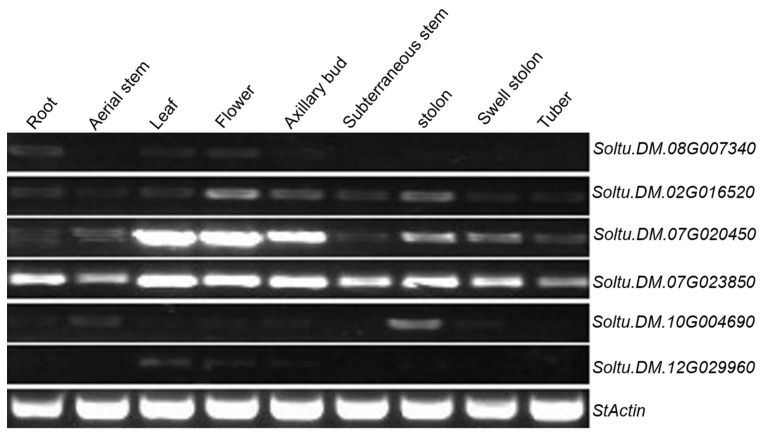
Semi-quantitative analysis of six *StTCP* genes with Stu-miR319 binding sites in different tissues of potato.

Numerous studies have demonstrated that several *TCP* genes contain miR319 binding sites. To analyze the binding site of miR319 to the *TCP* gene in potato, the online prediction website psRNATarget (http://www.zhaolab.org/psRNATarget/ (accessed on 20 February 2024)) was utilized. The analysis revealed that six *StTCP* genes possess binding sites for Stu-miR319, located at 1671–1691 bp, 1011–1032 bp, 1132–1153 bp, 1730–1759 bp, 1563–1583 bp, and 1419–1439 bp, respectively (Figure 4). To further explore which *TCP* gene among these six genes is more likely to be involved in potato growth and development, semi-quantitative experiments were conducted on various potato tissues, including root, aerial stem, leaf, flower, axillary bud, subterranean stem, stolon, swollen stolon, and tuber. The results showed that *StTCP10* (*Soltu.DM.07G023850*) exhibited the highest expression levels in stolon, swollen stolon, and tuber tissues compared to other *TCP* genes with Stu-miR319 binding sites (Figure 5). Given these results, *StTCP10* was selected for further research.

### 2.4. Acquisition and Identification of Transgenic Potato Plants

**Figure 6:**
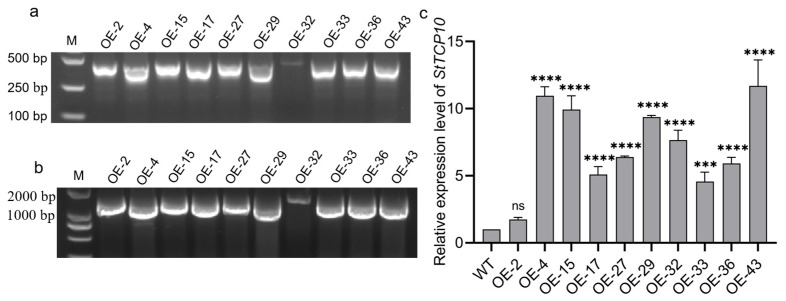
PCR detection with two pairs of primers in the regenerated plants and qRT-PCR analysis of *StTCP10* in the transgenic plants and wild type (WT). (**a**) Detection using *Kan*-specific primers, M: DL 2000 marker; (**b**) primer detection of *StTCP10* gene, M: DL 8000 marker; (**c**) qRT-PCR analysis of *StTCP10* in the transgenic plants and WT. The error line represents the standard deviation (*n* = 3); ns indicates no significant difference, *** *p* < 0.001, **** *p* < 0.0001.

To generate *StTCP10*-overexpressing potato plants, we constructed a CaMV 35S promoter driven overexpression vector and introduced it into tetraploid potato plants by *Agrobacterium*-mediated genetic transformation, yielding 56 regenerated potato plants. Transgenic lines were initially screened through PCR amplification of both the Kanamycin resistance gene (*Kan*) gene and partial *StTCP10* fragments from genomic DNA. Following PCR amplification with two pairs of primers, significant specific bands were observed (Figure 6a,b), consistent with the expected size, indicating that the overexpression vector of *StTCP10* had been transformed into potato plants.

Subsequently, the *StTCP10* expression levels in the abovementioned positive transgenic lines were analyzed via qRT-PCR. OE-2 showed comparable expression to wild-type plants, while the expression levels of *StTCP10* in the other transgenic plants were significantly higher than those in the wild-type plants (Figure 6c), demonstrating that the successful overexpression of *StTCP10* in the transgenic potato plants. Based on these results, OE-4 and OE-32 were selected for further experimentation.

### 2.5. Effect of StTCP10 Overexpression on Potato Tuber

**Figure 7:**
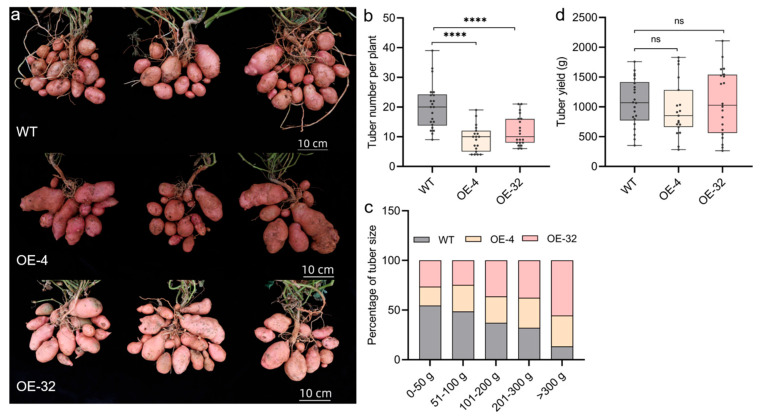
Effects of overexpression of *StTCP10* on tuber and yield in potato plants. (**a**) Phenotypes of transgenic plants and wild-type potato plants. For overexpression of transgenic plants, two lines are shown. Scale bar = 10 cm. (**b**) Comparison of the number of tubers per plant between WT plants and overexpressing plants. *n* > 19. (**c**) Distribution of tuber size in WT plants and overexpressing plants: 0–50, 51–100, 101–200, 201–300, and > 300 g. (**d**) Comparison of tuber yield per plant between WT plants and overexpressing plants. *n* > 19. The error line represents the standard deviation (*n* > 19); ns indicates no significant difference, **** *p* < 0.0001.

To determine the biological function of *StTCP10* in tuber development, the transgenic and wild-type potatoes were planted in fields for phenotypic observation. The tuber number, distribution of tuber size, and yield per plant were recorded and analyzed between transgenic potatoes and wild-type plants. Compared to the wild-type plants, the tuber number in the transgenic lines with *StTCP10* overexpression presented a significantly decreased phenotype, with a reduction of approximately 52% and 37%, respectively (Figure 7a,b). For tuber size distribution analysis, tuber size was categorized into five ranges: 0–50 g, 51–100 g, 101–200 g, 201–300 g, and >300 g. Compared to those of wild-type plants, the percentage of tuber sizes ranging from 0 to 50 g and 51 to 100 g was lower for the transgenic lines with *StTCP10* overexpression, whereas the percentage of tubers ranging from 101 to 200 g, 201 to 300 g, and > 300 g was greater than that for the wild-type plants (Figure 7c). However, no significant difference in the final potato yield was detected between the overexpressing plants and the wild type (Figure 7d). These results indicated that the *StTCP10* gene enhances tuber size and mediates the balance between tuber size and number in potato.

## 3. Discussion

Edible tubers are a striking feature of potato, with their yield determined by both tuber number and tuber size. Extensive evidence indicates TCP proteins, a class of plant-specific transcription factors, are critical regulators of plant growth and development and are extensively involved in the emergence and evolution of novel traits [4,13,14]. The role of TCP transcription factors in potato development has been partially elucidated. *StTCP1* modulates branching and promotes secondary tuber growth and enlargement [28,29], while *StTCP15* participates in potato tuber dormancy and germination by regulating the ratio of abscisic acid to gibberellin [30]. Furthermore, the *BRANCHED1a* (*BRC1a*) gene, a potato TCP, controls the growth of both aerial and underground lateral shoots [26], and BRC1b mutants exhibit ectopic aerial tubers and reduced underground tuberization [27]. Furthermore, *StTCP23*-silenced plants display stunted, curled leaves, and increased susceptibility to disease [31,32]. Despite these advances, the biological function of TCP transcription factors containing miR319 binding sites remains poorly understood in potato.

To address this question, we identified 32 *TCP* genes containing the conserved TCP domain through bioinformatics analysis, which were unevenly distributed across 12 chromosomes (Figure 1 and Figure 2) with encoded proteins varying in length from 157 to 577 amino acids (Table S1), suggesting potential structural and functional diversity among these transcription factors. This diversity provides a foundation for further in-depth studies on the specific biological functions of each *TCP* gene. Considerable work has demonstrated that several TCP family members are targeted by miR319 [34,35]. Our target analysis identified six *StTCP* genes with Stu-miR319 binding sites (Figure 4), implying that these genes may play significant roles in the growth and development of potato. Semi-quantitative experiments further revealed that *StTCP10* exhibited the highest expression levels in stolon, swollen stolon, and tuber tissues compared to other miR319 target genes (Figure 5), highlighting its potential importance in tuber development. To figure out the biological function of *StTCP10*, we constructed an overexpression vector and introduced it into potato plants by *Agrobacterium*-mediated genetic transformation, successfully generating transgenic lines with *StTCP10* overexpression (Figure 6). In tomato, LANCEOLATE (*LA*), a miR319-regulated CIN-class *TCP* gene, influences compound leaf formation [37]. *GhTCP4* is negatively regulated by miR319, leading to thinner cell walls in cotton fibers [39]. Additionally, miR319/TCP4 can affect tomato resistance to root knot nematodes by regulating the expression of jasmonic acid (JA) synthesis genes and the accumulation of endogenous JA [40]. *ApTCP2* in *Acer palmatum* regulates leaf morphology, flowering periods, and leaf senescence through miR319 [41]. These findings underscore the role of miR319/TCP interactions in plant development. Our field experiments demonstrated that *StTCP10* with Stu-miR319 binding sites significantly reduced tuber number (Figure 7b); on the other hand, the percentage of tubers sizes < 100 g was lower for transgenic lines with *StTCP10* overexpression, whereas the percentage of tubers ranging from 101 to 200 g, 201 to 300 g, and >300 g was greater than that for the wild-type plants (Figure 7c). These results suggest that *StTCP10* may promote tuber enlargement and development, leading to increased tuber size, while simultaneously inhibiting tuber differentiation, resulting in fewer tubers. However, although no significant difference in total yield was observed between overexpressing plants and wild-type plants (Figure 7d), a previous study revealed that tuber yield is fundamentally determined by tuber number and tuber size, which collectively govern final yield [2], so the balance between tuber number and size may explain the stable total yield observed in transgenic plants. Our findings indicate that *StTCP10* plays a pivotal role in tuber size and provides a foundation for further elucidating the mechanisms by which *TCP* genes with Stu-miR319 binding sites regulate tuber development. Future research should focus on unraveling the precise molecular mechanisms underlying the Stu-miR319/StTCP10 module and its role in fine-tuning potato tuber size.

## 4. Materials and Methods

### 4.1. Plant Materials and Growth Conditions

The tetraploid potato cultivar Désirée was used as the genetic background in this study. Potato plants were propagated and maintained in vitro by culturing single stem nodes on basal Murashige and Skoog (MS) media (Coolaber #PM10101) supplemented with 3% (*w*/*v*) sucrose at 22 °C under long-day (LD) conditions (16 h: 8 h, light: dark photoperiod), with a light intensity 200 μmol∙m^−2^∙s^−1^.

### 4.2. Identification of Potato TCP Genes

To identify *TCP* genes in potato, protein sequences of the *Arabidopsis TCP* gene family were retrieved from the TAIR database (https://www.arabidopsis.org), and potato genomic files were obtained from the Spud DB (https://spuddb.uga.edu/). The *Arabidopsis* TCP protein sequences were aligned with the potato whole-genome protein sequences using TBtools software (v2.210). The aligned sequences were then filtered using the NCBI Batch Web CD-Search Tool (www.ncbi.nlm.nih.gov/Structure/bwrpsb/bwrpsb.cgi (accessed on 1 February 2024)) to remove sequences lacking the conserved TCP domain. Additionally, the Pfam database (www. pfam.xfam.org) was used to screen and confirm the presence of the TCP domain (PF03634). Redundant sequences were removed, and the remaining sequences were designated as potato *TCP* genes.

### 4.3. Phylogenetic Analysis and Chromosomal Localization

A phylogenetic tree was constructed using the neighbor-joining (NJ) method in MEGA11 software, with Bootstrap values calculated from 1000 replicates. Chromosomal location information for the potato *TCP* genes was extracted from the potato genome and annotation files using TBtools software (v2.210), and a chromosomal localization map was generated.

### 4.4. Tissue-Specific Expression Profiling Analysis and Target Prediction Tool

To characterize the tissue-specific expression patterns of the *StTCP* genes, transcriptome data from the potato DM6.1 version was obtained from the Spud database. Expression data for *StTCP* genes were extracted, and FPKM (Fragments Per Kilobase of transcript per Million mapped reads) values in root, stem, leaf, shoot tip, stolon, young tuber, and mature tuber tissues were calculated. The FPKM values were then converted to log2FPKM and visualized as a heatmap using TBtools software. The psRNA Target online tool (https://www.zhaolab.org/psRNATarget/ (accessed on 5 February 2024)) was utilized to predict the target genes of potato miR319 within the *TCP* gene family.

### 4.5. Cloning of StTCP10 in Potato Tetraploid Désirée

In this study, the DM6.1 genome was used as a reference. Total RNA was extracted from Désirée plantlets, and cDNA was synthesized by reverse transcription following the manufacturer’s instructions. To clone the *StTCP10* (gene ID: Soltu.DM.07G023850), primers were designed based on the DM genome sequence, and the target gene was amplified using PCR. The PCR products were subsequently cloned into a cloning vector. Ten clones were randomly selected and validated through Sanger sequencing.

### 4.6. Construction of Overexpression Vector

To construct the overexpression vector, the cDNA fragment of *StTCP10* was inserted into the *Bam*HI and *Kpn*I restriction sites of the pCAMBIA1305 vector, generating the recombinant plasmid designated as *StTCP10*-1305. To facilitate genetic transformation, the *StTCP10*-1305 vector was digested with *Bam*HI and *Sac*I, and the resulting fragment was ligated into a pCAMBIA2301 vector with kanamycin resistance. The final construct, designated as *StTCP10*-2301, served as the overexpression vector for subsequent genetic transformation experiments.

### 4.7. Potato Genetic Transformation

The overexpression vector was introduced into *Agrobacterium tumefaciens* strain GV3101, which was then introduced into the tetraploid potato cultivar Désirée following a previously described method with slight modifications. Briefly, single stem nodes without axillary buds were excised from well-grown potato plants and placed on pre-culture medium (4.43 g/L MS basal salts, 30 g/L sucrose,8 g/L agar, 2 mg/L NAA, 1 mg/L 6-BA) for two days. The explants were then infected with *Agrobacterium* suspension (OD600 = 0.5) on a horizontal shaker at 50 rpm for 15 min at 22 °C. After infection, the explants were transferred to co-culture medium (identical to the pre-culture medium) and incubated at 22 °C for two days. Following co-culture, the explants were transferred to callus induction medium (CIM; 4.43 g/L MS basal salts, 30 g/L sucrose, 8 g/L agar, 2 mg/L ZT, and 400 mg/L cefotaxime) and cultured at 22 °C for 12 days. Subsequently, the explants were transferred to selection induction medium (SIM; CIM supplemented with kanamycin) and subcultured onto fresh SIM medium at 12-day intervals. Shoot formation was typically observed after approximately three rounds of subculture. Developing shoots were carefully excised and transferred to fresh medium for further growth.

### 4.8. Identification of the Transgenic Plants

Genomic DNA was extracted from putative transgenic potato plants using the CTAB method. Transgenic plants were identified with PCR amplification using primers specific to the screening marker gene (*Kan*) and the target gene (*StTCP10*). The PCR conditions were as follows: initial denaturation at 95 °C for 3 min; 25 cycles of denaturation at 98 °C for 10 s, annealing at 55 °C for 30 s, and extension at 72 °C for 60 s; followed by a final extension at 72 °C for 10 min. PCR products were resolved by electrophoresis on a 1% agarose gel. The sequences of primers used for vector construction and transgenic plant identification are provided in Table S2.

### 4.9. Analysis of the Transgenic Potato Plants via qRT-PCR

Leaves from 2-week-old overexpressing transgenic plants and wild-type plants were collected for total RNA extraction using the TaKaRa MiniBEST Plant RNA Extraction Kit (TaKaRa cat#9769; Takara, Otsu, Shiga, Japan). Reverse transcriptase PCR was conducted via ReverTra Ace^®^ qPCR RT Master Mix with a gDNA Remover Kit (TOYOBO cat#FSQ-301; Toyobo, Osaka, Japan) following the manufacturer’s instructions. Quantitative RT-PCR (qRT-PCR) was performed using GoTaq^®^qPCR Master Mix (Promega cat#A6001; Promega, Madison, WI, USA). *StActin* was used as an internal reference gene to normalize expression levels and calculate fold changes. Each sample was analyzed with three biological replicates and three technical replicates. The primers used for qRT-PCR are listed in Table S2.

### 4.10. Phenotypic Analysis of Transgenic Potato Plants

Overexpressing transgenic plants and wild-type plants were used as experimental materials. Ten-day-old in vitro potato plantlets were transplanted into seedling bags (12 cm diameter, one plant per pot) and grown in a climate chamber under long-day (LD) conditions for two weeks. After acclimatization, the plants were transferred to the field with a ridge height of 20 cm and planting intervals of 30 cm. The number of tubers per plant, tuber size (g), and tuber yield were recorded after three months of field growth. For statistical analysis, plants from two independent transformation events were used, with at least nineteen individual plants per transformation event. Significant differences between transgenic and wild-type plants were assessed using one-way ANOVA. The experimental data are presented as mean ± standard error.

## 5. Conclusions

The study characterized *StTCP10*, a TCP transcription factor harboring Stu-miR319 binding sites, which is expressed in stolon, swollen stolon, and developing tuber tissues. Through *Agrobacterium*-mediated genetic transformation, we generated *StTCP10*-overexpressing transgenic potato lines and demonstrated that overexpression of *StTCP10* decreased tuber number per plant while enhancing tuber size. In summary, this study revealed that *StTCP10* with Stu-miR319 binding sites enhances tuber size and mediates the trade-off between tuber size and number. These results provide novel genetic targets for molecular breeding programs aimed at tuber size improvement and valuable germplasm resources for developing cultivars suitable for the market and processing industry.

## Data Availability

All the data in the paper have been included as a part of the paper or as Supporting Information.

## Supplementary Materials

The following supporting information can be downloaded at: https://www.mdpi.com/article/10.3390/plants14091403/s1, Figure S1: The original electrophoretic gel images correspond to Figure 5 in the main article; Figure S2: The original electrophoretic gel images correspond to Figure 6a and Figure 6b in the main article title; Table S1: TCP loci and encoded protein length in potato; Table S2: Primer sequences in this study.
