# Supplementary material for: Overexpression of StTCP10 Alters Tuber Number and Size in Potato (Solanum tuberosum L.)
Source: Plants (Basel). 2025 May 7;14(9):1403. doi: 10.3390/plants14091403 (PMC12073833; doi:10.3390/plants14091403)
Supplement: Supplementary file 1 [file plants-14-01403-s001.zip › Supplementary Files/Table S1 and Table S2.pdf]

Table S1 **TCP loci and encoded protein length in potato**

Gene ID	Chromosome	Start	End	Strand	Protein Length
Soltu.DM.01G003810.1	chr01	3900839	3901579	-	246
Soltu.DM.01G032640.1	chr01	72435473	72437381	+	280
Soltu.DM.01G042700.1	chr01	81053907	81056491	-	534
Soltu.DM.02G009190.1	chr02	24066430	24069201	-	222
Soltu.DM.02G010940.1	chr02	25740920	25741759	+	279
Soltu.DM.02G016520.1	chr02	31017582	31019055	-	334
Soltu.DM.02G029980.1	chr02	42428074	42429093	+	238
Soltu.DM.02G030790.2	chr02	43053201	43055134	-	283
Soltu.DM.03G006350.1	chr03	9481592	9482065	-	157
Soltu.DM.03G016530.1	chr03	40751778	40753787	-	366
Soltu.DM.03G016540.1	chr03	40758966	40759778	-	270
Soltu.DM.03G016550.1	chr03	40766767	40767504	-	245
Soltu.DM.03G029160.1	chr03	53668132	53669981	-	344
Soltu.DM.03G030410.1	chr03	54904362	54906342	+	414
Soltu.DM.03G034300.2	chr03	57733345	57735187	+	336
Soltu.DM.04G001360.1	chr04	1397253	1398855	-	381
Soltu.DM.04G005640.1	chr04	5967103	5967900	+	265
Soltu.DM.05G002510.2	chr05	1948044	1949348	-	348
Soltu.DM.05G005900.1	chr05	5187590	5189195	+	371
Soltu.DM.06G020280.1	chr06	46943149	46945175	+	327
Soltu.DM.06G025210.1	chr06	51156913	51158738	+	364
Soltu.DM.06G025480.1	chr06	51387826	51389321	-	266
Soltu.DM.06G026030.1	chr06	51895593	51897782	+	410
Soltu.DM.07G020450.1	chr07	50947017	50949690	-	311
Soltu.DM.07G023850.1	chr07	53797769	53800392	+	400
Soltu.DM.08G007340.1	chr08	13477139	13480497	+	342
Soltu.DM.08G007360.1	chr08	13629372	13632185	+	425
Soltu.DM.08G026800.1	chr08	56298254	56299810	+	374
Soltu.DM.09G005570.1	chr09	5120957	5121966	+	201
Soltu.DM.10G004690.1	chr10	4088030	4089763	+	577
Soltu.DM.11G012310.1	chr11	14497763	14499355	-	389
Soltu.DM.12G029960.1	chr12	59312362	59314529	-	421

Table S2 **Primer sequences in this study**

Primer Name	Primer Sequence(5'→3')
Kan-3F	GCACAATCCCACTATCCTTCG
Kan-3R	TCCCGCTTCAGTGACAACG
StActin-QF	CAAGTTATTACCATTGGTGCTGAGA
StActin-QR	TGCAGCTTCCATACCAATCATG
StTCP10-35BF	AACGGGGGATCTACCGGATCCATGGGAGAAACGTCAAC
StTCP10-KIR	ATGGTCTTTGTAGTCATGGCGAGAATCAGAG
StTCP10P-1F	GAACAGTAATGCCTCCTCACC
StTCP10P-1R	TATGAGCAGAGAGACGCACAC
StTCP10P-2F	CCTTGTAAGGGTACTATGGTAGG
StTCP10P-2R	TTTTCCACGCAATGTTGC
StTCP10-SF	AAGAAGAGGAGCACGATGG
StTCP10-SR	GCCAAACACATAACACTCCTAA
StTCP10-1F	TCTTCATTGGGGCAAAACG
StTCP10-1R	TCATTCTCCATCTCCAGGCTC
StTCP10-2F	ATGGGAGAAACGTCAAGATTG
StTCP10-2R	AGATCAAATTTTTGGAGCTAATTC
StTCP10-QF	TGGATTTGCCTCTGAAGTAGG
StTCP10-QR	TATCGGAAATGCCATCGTG
StTCP10-F	ATGGGAGAAACGTCAAGATTGG
StTCP10-R	TCAATGGCGAGAATCAGAGG
