# Supplementary figures and images for: Overexpression of StTCP10 Alters Tuber Number and Size in Potato (Solanum tuberosum L.)
Source: Plants (Basel). 2025 May 7;14(9):1403. doi: 10.3390/plants14091403 (PMC12073833; doi:10.3390/plants14091403)

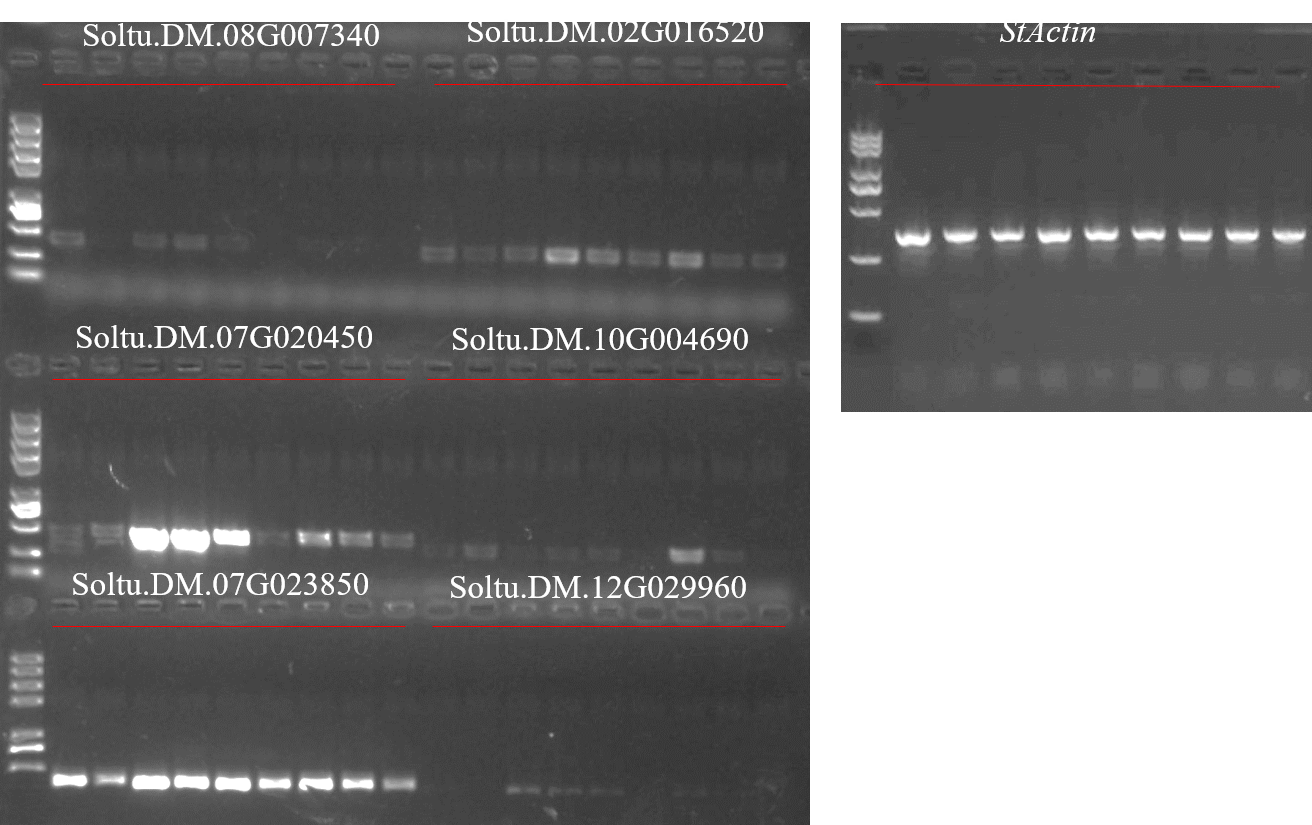

Supplement: Supplementary file 1 [file plants-14-01403-s001.zip › Supplementary Files/Figure S1.tif]

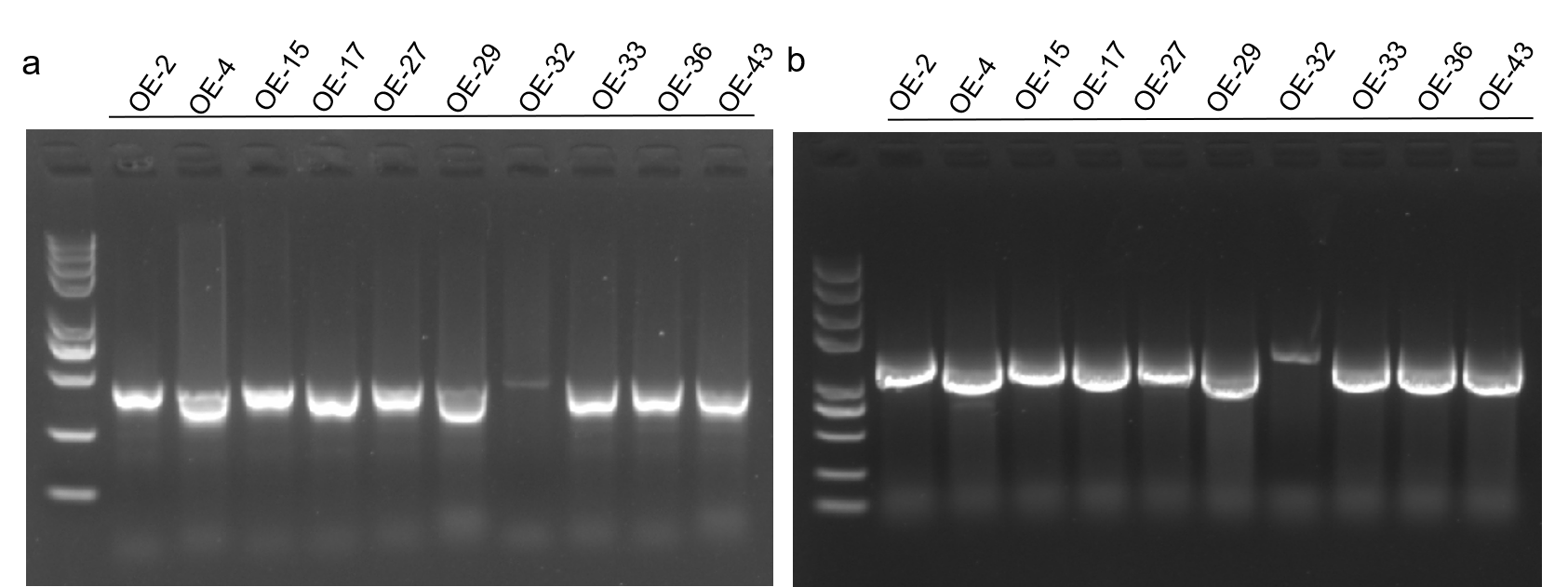

Supplement: Supplementary file 1 [file plants-14-01403-s001.zip › Supplementary Files/Figure S2.tif]
